# High Performance Solid Polymer Electrolytes for Rechargeable Batteries: A Self‐Catalyzed Strategy toward Facile Synthesis

**DOI:** 10.1002/advs.201700174

**Published:** 2017-08-02

**Authors:** Yanyan Cui, Xinmiao Liang, Jingchao Chai, Zili Cui, Qinglei Wang, Weisheng He, Xiaochen Liu, Zhihong Liu, Guanglei Cui, Jiwen Feng

**Affiliations:** ^1^ Qingdao Industrial Energy Storage Research Institute Qingdao Institute of Bioenergy and Bioprocess Technology Chinese Academy of Sciences Qingdao 266101 P. R. China; ^2^ Key Laboratory of Nanomaterials Qingdao University of Science and Technology Qingdao 266042 P.R. China; ^3^ State key Laboratory of Magnetic Resonance and Atomic and Molecular Physics Wuhan Institute of Physics and Mathematics Chinese Academy of Sciences Wuhan 430071 P. R. China; ^4^ University of Chinese Academy of Sciences 100049 Beijing P. R. China

**Keywords:** cationic mechanism, crosslinking, in situ polymerization, poly(ethylene glycol) diglycidyl ether, solid polymer electrolytes

## Abstract

It is urgent to seek high performance solid polymer electrolytes (SPEs) via a facile chemistry and simple process. The lithium salts are composed of complex anions that are stabilized by a Lewis acid agent. This Lewis acid can initiate the ring opening polymerization. Herein, a self‐catalyzed strategy toward facile synthesis of crosslinked poly(ethylene glycol) diglycidyl ether‐based solid polymer electrolyte (C‐PEGDE) is presented. It is manifested that the poly(ethylene glycol) diglycidyl ether‐based solid polymer electrolyte possesses a superior electrochemical stability window up to 4.5 V versus Li/Li^+^ and considerable ionic conductivity of 8.9 × 10^−5^ S cm^−1^ at ambient temperature. Moreover, the LiFePO_4_/C‐PEGDE/Li batteries deliver stable charge/discharge profiles and considerable rate capability. It is demonstrated that this self‐catalyzed strategy can be a very effective approach for high performance solid polymer electrolytes.

## 1 Introduction

Rechargeable lithium ion batteries using nonaqueous liquid electrolytes encounter severe thermal runaway, especially when they are packed into a large format battery module. These bottleneck problems are spurring great interests in the pursuit of high safety and high energy solid state lithium batteries.^1^ Solid state electrolyte is the critical component targeting high safety and high voltage window. Among all the reported solid state electrolytes, solid polymer electrolytes (SPE) offer outstanding advantages of high flexibility, easy processability, and low interfacial resistance over inorganic ceramic electrolytes. These merits qualify SPE intriguing materials for large format and high energy solid state batteries.^2^

Solid polymer electrolytes generally consist of a polymer host as solid matrix and a disassociated lithium salt as lithium ion conductor.^3^ The polymer host should have a low glass transition temperature facilitating chain segments mobility and a high dielectric constant dissociating enough lithium salts. In addition to the classic poly(ethylene oxide) PEO host polymer,^4^ polysiloxane,^5^, ^6^ and polycarbonate^7^, ^8^ have recently been demonstrated to serve as alterative host polymers with regard to their higher electrochemical stability. As to another component lithium salts, it includes lithium bis(trifluoromethanesulfonyl)imide (LiTFSI), lithium perchlorate (LiClO_4_), lithium bis(oxalato)borate (LiBOB), lithium difluoro(oxalate)borate (LiDFOB), lithium hexafluorophosphate (LiPF_6_), lithium tetrafluoroborate (LiBF_4_), and so on.^9^ Most of the lithium salts are composed of a simple anion stabilized by a Lewis acid agent and have a structure in which the formal negative charge is well delocalized by the Lewis acid ligands. It makes the corresponding complex salts lower melting point and better solubility in low dielectric media than their parent salts.^10^

Solid polymer electrolytes generally suffer from complicated and tedious preparation processes. The commonly used solution casting methods consumed a large amount of organic solvents for dissolving polymer hosts and lithium salts. In addition, the film‐casting and solvent evaporation process was relative expensive and unfriendly to environment as well. A solvent‐free hot‐pressing method was developed to fabricate the crosslinked PEO electrolyte membrane.^11^ However, it was still an ex situ process and required hot‐pressing machines. It is noted that most researches focus on aspects of synthesizing novel polymer hosts with suitable ionic conductivity or electrochemical compatibility with high voltage cathodes.^12^ However, few attention has ever been paid to the processing method.^13^ So, it is urgent to explore high performance solid polymer electrolytes via a simple chemistry route.^14^ Recently, in situ generation of high performance solid polymer electrolytes has been developed in our group. In the process of in situ polymerization for preparing solid polymer electrolyte, the liquid monomers or precursors are injected into the batteries enabling an excellent contact and affinity between the polymer electrolyte and cathodic/anodic electrodes. This cost‐effective and readily scalable strategy is propitious to the preparation of solid polymer electrolyte. However, the free radical polymerization reaction of unsaturated carbon–carbon double bonds always requires addition of a thermal and UV‐light initiator. This radical polymerization between confined electrodes is not reliable due to lack of reproducibility caused by the interfacial side reactions.^15^, ^16^ Therefore, initiator‐free and reliable in situ synthetic chemistry strategy is highly desirable.

The above‐mentioned lithium salts are based on complex anions that are composed of a simple anion stabilized by a Lewis acid agent. The anion of LiBF_4_ could be viewed as F complexed by the Lewis acid BF_3_, which was also known as anions of super acids.^17^ Miwa et al. reported polymerization of bis‐oxetanes using lithium salts as initiators (including LiBF_4_, LiPF_6_, LiN(CF_3_SO_2_)_2_, and LiN(C_2_F_5_SO_2_)_2_), indicating that the self‐catalyzed strategy might be an effective approach for preparing solid polymer electrolytes.^13^ Lithium difluoro(oxalato)borate (LiDFOB) is thermally much stable in pure solid state. However, LiDFOB undergoes some trace disproportionation reaction at elevated temperatures producing lithium tetrafluoroborate (LiBF_4_) and lithium bis(oxalato)borate (LiBOB).^18^ The poly(ethylene glycol) diglycidyl ether (PEGDE) has high activity due to the two epoxy groups, which can be employed for crosslinking reactions to form polymer network. In addition, the epoxy ring can be chemically opened via a cationic polymerization reaction in the present of Lewis acid. Moreover, the crosslinking method is considered to be a simple and effective way to suppress the crystallization and to enhance the mechanical strength.^19^, ^20^ Herein, a self‐catalyzed strategy toward crosslinked SPEs has been presented, providing a simplified assembly process of all‐solid state LIBs.

## 2 Results and Discussion

**Figure 1:**
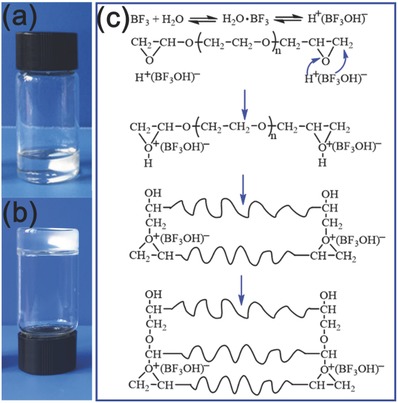
a) The optical images of PEGDE solution with LiDFOB and b) crosslinked solid electrolyte pure C‐PEGDE; c) the cationic polymerization mechanism initiated by BF_3_.

Liquid PEGDE can be polymerized into pure C‐PEGDE initialized by addition of LiDFOB at 80 °C for 4 h (shown in **Figure** **1**a,b). The reaction mechanism can be explained as a cationic polymerization process initiated by trace BF_3_, resulted from thermodecomposition of LiDFOB under heating conditions.^17^, ^20^, ^21^ The cationic polymerization mechanism is presented in Figure 1c. BF_3_ can be activated with the trace moisture to form H^+^(BF_3_OH)^‐^, which act as a more efficient cationic polymerization initiator to promote the ring opening reaction. During the initiation of the reaction, H^+^(BF_3_OH)^‐^ attack the epoxy group and loosen up the epoxy bonds to form initiating complex, and subsequently the propagation step proceeds.^22^ Figure S2 (Supporting Information) showed the typical image of in situ polymerization of PEGDE into pure C‐PEGDE initiated by LiBF_4_, LiClO_4_, and LiPF_6_ heated at a temperature of 80 °C within a few hours. However, the reaction could not be initiated by LiTFSI and LiBOB at a temperature of 80 °C within 20 h. Compared to the frequently used methods in the polymerization reaction of SPEs, the fabrication of C‐PEGDE system possesses huge advantages, including free of additional catalyst or initiator, in situ synthesis, and the resultant poly (ethylene oxide) (PEO) chains. Free of additional catalyst or initiator and lithium salts as initiator would suppress the side reaction and enable the SPEs possess chemical stability during the ionic conductivity. In situ synthesis method greatly simplified the assembly process of the all‐solid state LIBs compared with an ex situ method. The SPEs composed of resultant PEO chains would maintain a high dielectric constant and strong Li^+^ solvating ability. Therefore, this facile in‐situ synthetic chemistry strategy via a cationic polymerization mechanism is efficient and reliable for preparing high performance polymer electrolytes.

To identify the initiation system of the reaction, Fourier transform infrared spectroscopy (FTIR) and solid state NMR measurements were conducted to analyze the chemical structure of PEGDE and pure C‐PEGDE. Figure S1 (Supporting Information) showed the FTIR spectrums of PEGDE, the composite solution and pure C‐PEGDE solid electrolyte. The peaks around 2875, 1454, and 1350 cm^−1^ were attributed to the C—H stretching, asymmetric stretching and bending vibration in —CH_2_ and —CH, respectively.^23^ The appearance of peaks at 1110 cm^−1^ belongs to the C—O—C stretching. After the addition of LiDFOB, two prominent feature peaks appear at 1798 and 1759 cm^−1^, which were assigned to C=O oscillating in phase and out of phase, respectively.^24^ The epoxy groups present a weak absorption peak at 911 cm^−1^ in the PEGDE, which almost disappears in the pure C‐PEGDE solid electrolyte. While, there is an increase in the intensity of C–O–C absorption peak at 1110 cm^–1^ in the pure C‐PEGDE. These results demonstrate that the epoxy groups are ring opened. In addition, in the spectrum of pure C‐PEGDE, the peak at 1798 cm^−1^ shifts to 1740 cm^−1^, which indicates that the LiDFOB has an interaction with PEGDE.^21^, ^22^

**Figure 2:**
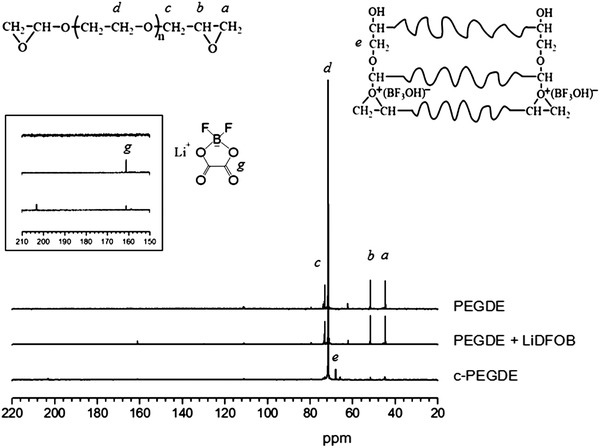
^13^C MAS NMR spectra for PEGDE, PEGDE with LiDFOB solution before heating, and the pure C‐PEGDE. The spectra were measured by one pulse sequences with high power decoupling for ^1^H at 11.7 T.

**Figure** **2** showed the ^13^C magic angel spinning nuclear magnetic resonance (MAS NMR) results for PEGDE with LiDFOB systems, providing valuable structure information of carbon skeleton of the polymers. The peak at 161 ppm from the carbonyl group of LiDFOB was about 25% remaining after polymerization. The other 75% was shifted to 159 ppm as a broaden peak attributed to the salt that participated in the polymerization processes. This indicated that the carbonyl group was likely linked to the end of the chain. The 73.4 ppm peak was assigned to the methylene (—CH_2_—) which linked the end epoxy groups and the main chain. The 51.5 and 44.6 ppm peaks were from the tertiary carbon (—CH—) and secondary carbon (—CH_2_—) of the terminal ring groups, respectively. The proportion of opened rings versus the original rings could be obtained by comparing the integral peak area after the polymerization. In this LiFDOB system, there was about 90% ring opened. The small signal of 62 ppm was ascribed to the trace carbon of terminated —CH_2_—OH in PEGDE, which disappeared after the reaction. After polymerization, there were two peaks arising at 68 and 66 ppm. The 68 ppm resonance was from the methylene (—CH_2_—) linking two PEGDE reactant chains in the crosslinked frameworks. The other peak of 66 ppm was assigned to the carbon connecting the skeleton and terminal group hydroxyl (—CH_2_—OH). Besides, the resonance of 70.9 ppm from the main chain (—CH_2_—CH_2_—O—) was broadened after polymerization, indicating that it was a solidified or crosslinking process when compared with the solution state.

**Figure 3:**
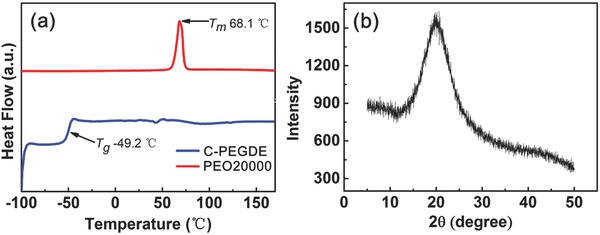
a) DSC profiles of pure C‐PEGDE solid electrolyte and PEO20000; b) XRD patterns of pure C‐PEGDE solid electrolyte.

The amorphous phase is in favor of better ionic conductivity because Li‐ions transport fast in the amorphous region of the polymer electrolyte. To explore the crosslinking effect of pure C‐PEGDE after polymerization, the differential scanning calorimetry (DSC) was conducted to investigate the thermal behavior. As shown in **Figure** **3**a, two characteristic endothermic peak at −49.2 and 68.1 °C were observed in pure C‐PEGDE solid electrolyte and PEO20000, respectively. They were assigned to the glass transition temperature (*T*_g_) of pure C‐PEGDE and the melting point (*T*_m_) of PEO2000, respectively. The pure C‐PEGDE presented no melting point because of the crosslinked framework inhibiting crystallization. There was no crystallization peak in X‐ray diffraction pattern (Figure 3b), which agreed well with the result of DSC. The amorphous pure C‐PEGDE can be a favorable medium for ions transportation. These intrinsic amorphous nature and low *T*_g_ would be beneficial for high ionic conductivity.

**Figure 4:**
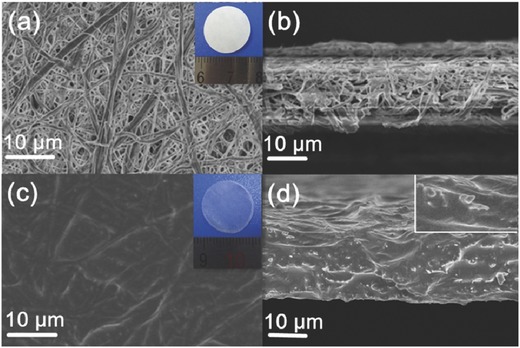
Typical SEM images of a) cellulose membrane surface (inset shows the photograph of cellulose membrane) and b) cross‐section; SEM images of c) composite membrane surface (inset shows the photograph of composite membrane), and d) cross‐section (inset shows the magnified SEM image).

A nonwoven cellulose membrane was used as a self‐standing substrate to prepare composite C‐PEGDE SPE. To visualize the structure of the composite C‐PEGDE polymer matrix in SPE, Scanning Electron Microscope (SEM) images of the surface and cross‐section of cellulose and composite membrane were observed. An obviously porous network of pristine cellulose membrane was seen as shown in **Figure** **4**a. Inset of Figure 4a shows the photograph of cellulose membrane. The pores of cellulose membrane were randomly arranged with an average diameter size of 800 nm and large‐sized pores exceeded 2 µm. This was a favorable framework for mechanically supporting polymer electrolyte.^25^ Generally, membrane matrix with a large pore size is not beneficial to low self‐discharging. After compositing with PEGDE by in situ polymerization, the pores were incorporated with C‐PEGDE polymer. A dense and continuous polymer phase was formed inside the membrane. Cellulose‐supported composite solid polymer electrolyte with a continuous phase was of great benefits to block the growth of lithium dendrites and to prevent microshort circuiting (Figure 4c).^26^ In addition, the cross‐section image showed that the in situ generated C‐PEGDE solid polymer electrolyte uniformly incorporated into the pores of cellulose membrane generating continuous channels which was in favor of lithium ions transportation (Figure 4d).^25^ The thickness of the composite membrane was about 30 ± 5 µm. Inset of Figure 4c shows the composite C‐PEGDE cellulose membrane was transparent, flexible, and robust.

**Figure 5:**
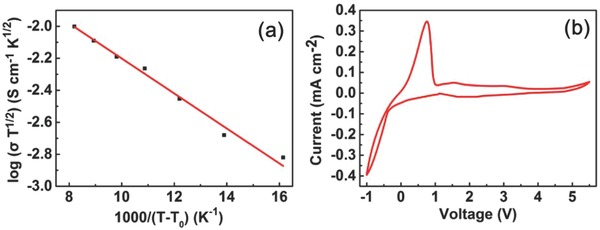
a) The temperature dependence of ionic conductivity for the composite C‐PEGDE polymer electrolytes; b) the electrochemical stability window of the composite C‐PEGDE electrolyte.

**Figure** **5**a presented the temperature dependent ionic conductivity for the composite C‐PEGDE polymer electrolyte over the range of temperature from 298 to 353 K. The ionic conductivity of composite C‐PEGDE is 8.9 × 10^−5^ S cm^−1^ at ambient temperature, which is close to pure C‐PEGDE, but higher than that of previous reported PEO/LiTFSI‐based electrolyte 5 × 10^−5^ S cm^−1^.^27^ The enhanced ionic conductivity may be due to the crosslinking reaction suppressing the crystallization of the PEO segments. It could be observed that the ionic conductivity agreed well with the Vogel–Tamman–Fulcher empirical equation.^28^ Figure 5b depicted the electrochemical stability window of the composite C‐PEGDE electrolyte. The anodic current onset was related to the electrochemically oxidized decomposition of polymer electrolyte. In the negative scan, a high current was observed from about −0.20 V versus Li/Li^+^, corresponding to the lithium plating on the stainless steel electrode. In the positive scan, a current peak of lithium stripping appeared at about 0.5 V versus Li/Li^+^. There were no obvious decomposition currents up to 4.5 V versus Li/Li^+^, indicative of meeting the electrochemical stability requirements for LIBs.

**Figure 6:**
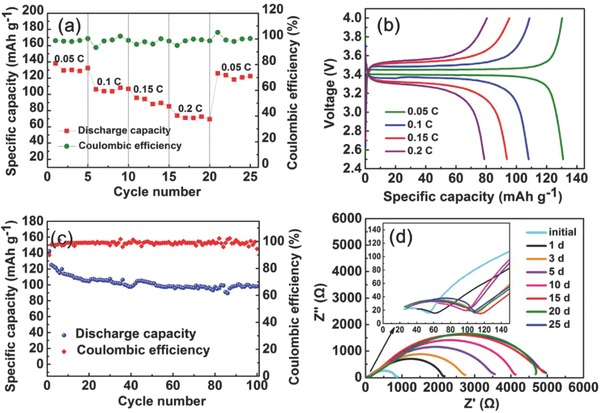
a) Rate capability of LiFePO_4_/C‐PEGDE/Li cells using the composite C‐PEGDE at ambient temperature; b) the charge/discharge curves of LiFePO_4_/C‐PEGDE/Li cells using the composite C‐PEGDE at varied current densities; c) cycling performance of the LiFePO_4_/C‐PEGDE/Li cells at a current density of 0.1 C within the voltage range of 2.5–4.0 V; d) time evolution of the interfacial resistance of Li/C‐PEGDE SPE/Li using in situ polymerized composite C‐PEGDE.

The solid state LiFePO_4_/C‐PEGDE/Li cells were assembled to evaluate the electrochemical performance of composite C‐PEGDE SPE in LIBs. The rate capability of the LiFePO_4_/Li cells with composite C‐PEGDE SPE at various rates from 0.05 to 0.2 C at room temperature is shown in **Figure** **6**a and the corresponding charge/discharge profiles are shown in Figure 6b. The reversible capacities of composite C‐PEGDE SPE were about 135, 115, 100, and 80 mAh g^−1^ at the discharging rate of 0.05, 0.1, 0.15, and 0.2 C, respectively. The LiFePO_4_/Li cells showed a typical voltage plateau at 3.5 and 3.4 V, which was due to the Li/Li^+^ redox reaction of LiFePO_4_. Figure 6c showed the specific discharge capacity of LiFePO_4_/Li cells at ambient temperature. After 100 cycles at 0.1 C, the LiFePO_4_/Li cell delivered a discharge capacity of 95 mAh g^−1^ and the capacity retention ratio was about 74.2%. The alternating current impedance spectra of Li/C‐PEGDE SPE/Li were shown in Figure 6d. There were two semicircles in the impedance spectrum, which was due to the bulk electrolyte resistance and interfacial resistance between lithium electrode and polymer electrolyte. No obvious change of bulk electrolyte resistance was found during the measuring process. The interfacial resistance between lithium electrode and polymer electrolyte continually increased to some extent during the first 20 d, then kept quite stable after the 20th day. It was consistent with that the composite C‐PEGDE‐based electrolyte exhibited stable charge/discharge process after the 20 cycles (shown in Figure 6c).

**Figure 7:**
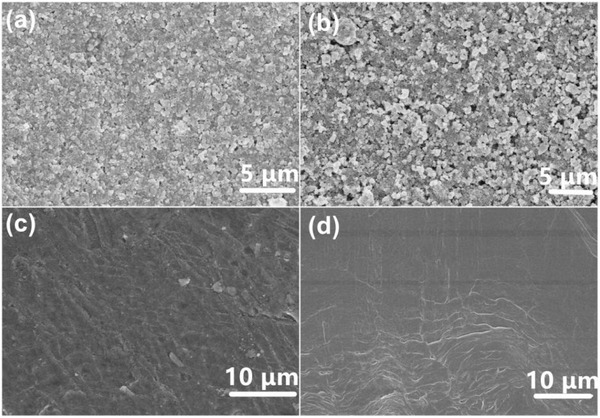
Typical SEM images of a) the LiFePO_4_ cathode after 100 cycles and b) the pristine LiFePO_4_; typical SEM images of c) lithium metal foil after 100 cycles and d) the pristine lithium metal foil.

To gain insight into the positive effect of composite C‐PEGDE on battery performance, the surface morphology of LiFePO_4_ cathode and lithium foil anode were observed by SEM after 100 cycles, which are presented in **Figure** **7**. Some polymer electrolyte was incorporated into the porous cathodes originated from the in situ polymerization process when compared with that of the pristine LiFePO_4_ cathode (Figure 7b). These results manifested that close contact between the SPE and cathode interface would be greatly beneficial for improving the electrolyte/electrode interfacial contacts and mitigating the interfacial impedance. It was shown in Figure 7c that the surface of lithium anode became slightly rough. There was a compact solid electrolyte interfacial (SEI) layer on the surface of lithium anode and no apparent dendrites or defects were observed after 100 cycles compared with the pristine lithium metal foil. Generally, uneven electrodeposition exacerbates dendritic structure by creating fresh surface for additional reactions. Therefore, the relatively smooth surface of lithium after long cycles was attributed to the homogenous composite C‐PEGDE solid electrolyte resulting in uniform lithium deposition/striping. Figure S3 (Supporting Information) presented Chronopotentiometry results of Li/C‐PEGDE SPE/Li symmetrical cells at room temperature at the current density of 0.02 mA cm^−2^. There was no observable short circuit phenomenon after 400 h polarization at 0.02 mA cm^−2^ and the surface of lithium anode became slightly rough. The sufficient rigidity of solid state composite C‐PEGDE would suppress dendrites crossover during a long‐term cycle life and prevent short circuit occurrence, indicating a good compatibility between composite C‐PEGDE and lithium anode. As shown in the Figure S4 (Supporting Information), the equivalent circuit presented a semicircle of impedance spectra in the high frequency range and a semicircle in the medium‐to‐low frequency region. The first semicircle was assigned to the diffusion resistance of Li^+^ ions through the SEI film deposited on the electrode (*R*_SEI_). The second semicircle was owing to the electrochemical reaction resistance (*R*_e_ and *R*_ct_). For C‐PEGDE‐based LiFePO_4_/Li cells, *R*_SEI_ increased from 202.3 to 230.7 Ω and electrochemical reaction resistance (*R*_e_ + *R*_ct_) increased from 1316.05 to 5974 Ω. It was demonstrated that the initial capacity fading within the first 20 cycles was attributed to the increasing electrochemical reaction resistance (*R*_e_ + *R*_ct_).

## 3 Conclusion

We have prepared a crosslinked poly(ethylene glycol) diglycidyl ether‐based electrolyte by a self‐catalyzed strategy and in situ technology via a facile cationic polymerization initiated by lithium salts. The composite C‐PEGDE SPEs possessed excellent ionic conductivity, cycling behavior, and C‐rate capability thanks to the amorphous phase of crosslinked structure. These results demonstrated that this facile and reliable in situ synthetic chemistry strategy via a cationic mechanism was very promising to prepare high performance polymer electrolytes.

## 4 Experimental Section

*Synthesis of C‐PEGDE Solid Electrolyte Films*: PEGDE (average molecular weight 500, water content 556 ppm, Maklin), lithium bis(trifluoromethane sulfonimide) (LiTFSI, Maklin), lithium hexafluorophosphate (LiPF_6_, Energy Chemical), lithium difluoro(oxalato)borate (LiDFOB, DoDoChem), lithium bis(oxalate) borate (LiBOB, DoDoChem), lithium perchlorate (LiClO_4_, Maklin), and lithium tetrafluoroborate (LiBF_4,_ Maklin) were battery grade. The cellulose membrane was purchased from Japanese NKK (TF4030) with thickness of about 25 µm. The PEGDE monomer was dried by using 3 Å molecular sieve and the contents of trace water in PEGDE were detected by the Karl Fischer method (831 KF Coulometer, Metrohm). Due to the hygroscopic nature of these compounds, all regents for preparing the SPE were stored in an argon‐filled glove‐box.

The solutions of the optimized composition by adding 0.4 mmol lithium salt (LiPF_6_, LiDFOB, LiBOB, LiClO_4_, LiBF_4_, and LiTFSI) into 2 mL PEGDE respectively with a magnetic stirrer stirred continuously at room temperature for a few hours in order to obtain homogeneous solutions. Then the homogeneous mixture precursor solution was heated at a temperature of 80 °C for a few hours to obtain the solid crosslinking polymer electrolyte.

Composite C‐PEGDE (crosslinked PEGDE) solid electrolyte films were synthesized by in situ thermal polymerization of a precursor inside the pores of cellulose membrane. A homogeneous and transparent solution of 1.25 wt% LiDFOB and 20 wt% LiTFSI involved in PEGDE was injected into a test cell with a cellulose separator. After that, the test cells were heated at 80 °C for 4 h to ensure the PEGDE polymerized into pure C‐PEGDE, The calculated polymerization conversion of PEGDE into C‐PEGDE was more than 90%.

The cathode was prepared by blending 80 wt% active material (LiFePO_4_), 10 wt% electronic conductor (super P carbon black), and 10 wt% binder (LA133) with appropriate deionized water and then casting on the aluminum foil to be dried under high vacuum at 120 °C for 12 h. The mass loading of the LiFePO_4_ was about 3.6 mg cm^−2^. The LiFePO_4_/C‐PEGDE SPE/Li coin cells were in situ assembly in an argon‐filled glove box by adding the precursors (20 wt% LiTFSI dissolved in PEGDE) into the CR2032‐type coin cells with cellulose separator as supporting matrix. After that, all the assembled cells kept standing for 4 h to completely wet the electrolyte/electrode interface, then heated to 80 °C for 4 h to ensure the PEDGE monomer polymerized and crosslinked.

*Chemical Characterization of the Pure C‐PEGDE*: The FTIR spectra of the pure C‐PEGDE solid electrolyte were characterized using a Frontier FTIR spectrometer (Perkin‐Elmer) in the range of 4000–400 cm^−1^. ^13^C solid state NMR experiments were performed on a Bruker Avance III 500 MHz NMR spectrometer (11.7 T) with a 4 mm probe at Larmor frequencies of 125.9 MHz. The ^13^C chemical shifts were referenced to a solid external reference adamamtane as 39.0 ppm. ^13^C MAS NMR spectra were acquired at a spin rate of 5 kHz with high power decoupling for proton and a long enough recycle delay to assure complete relaxation. X‐ray diffraction (XRD) data were obtained with a Bruker‐AXS Microdiffractometer (D8 Advance) using Cu *K*_α_ radiation (λ = 1.5406 Å). Differential scanning calorimetry of pure C‐PEGDE was conducted on DSC 200F3 (Netzsch) from −100 to 200 °C. The thermogravimetric analysis was test in argon atmosphere using a Mettler‐Toledo thermogravimeter (1100SF) in the temperature range of 50–600 °C at a heating rate of 10 °C min^−1^.

*Electrochemical Measurements*: The ionic conductivities of the composite C‐PEGDE were analyzed by conducting AC impedance spectroscopy (Zahner‐elektrik IM6E) using a test cell consisting of a small piece of SPE film sandwiched between two stainless steel blocking electrodes at a frequency range of 0.1 Hz to 1 MHz. The AC impedance measurements were carried out with amplitude of 5 mV at temperatures ranging from 20–80 °C by an electrochemical workstation (Shanghai Chenhua CHI660C). The ionic conductivity of polymer electrolyte was calculated by using following equation

(1) $$\sigma \;\;=\;\;d\;\times \;s^{-1}\;\times \;R_{b}^{-1}$$

where σ presents the ionic conductivity, *d* is the thickness of the solid polymer electrolyte, *R*_b_ corresponds to the bulk resistance of polymer electrode, and *s* is the effective contact area between the electrode and electrolyte.

The cycle voltammetry (CV) curves were recorded from −1.0 to 5.5 V versus Li/Li^+^ at a scanning rate of 0.1 mV s^−1^ using a stainless‐steel as the working electrode and lithium metal as the counter and reference electrode by a electrochemical workstation.

The interface resistance between the composite C‐PEGDE and the lithium metal electrode was measured by electrochemical impedance spectroscopy (EIS) with an Li/C‐PEGDE/Li symmetrical cell from 100 to 4 MHz for different aging time at 25 °C.

The charge–discharge and cycling performance was test using a Land battery test system between 2.5 and 4.0 V at 25 °C with a rate of ranging from 0.02 to 0.2 C. The EIS of batteries after three cycles and 100 cycles were measured from 100 mHz to 4 MHz with applied voltage amplitude of 5 mV.

## Conflict of Interest

The authors declare no conflict of interest.

## Supporting information

Supplementary
