# Supplementary material for: High Performance Solid Polymer Electrolytes for Rechargeable Batteries: A Self‐Catalyzed Strategy toward Facile Synthesis
Source: Adv Sci (Weinh). 2017 Aug 2;4(11):1700174. doi: 10.1002/advs.201700174 (PMC5700653; doi:10.1002/advs.201700174)
Supplement: Supplementary file 1 — Supplementary [file ADVS-4-na-s001.pdf]

Copyright WILEY-VCH Verlag GmbH & Co. KGaA, 69469 Weinheim, Germany, 2017.

ADVANCED SCIENCE Open Access

Supporting Information

for *Adv. Sci.,* DOI: 10.1002/advs.201700174

High Performance Solid Polymer Electrolytes for Rechargeable Batteries: A Self-Catalyzed Strategy toward Facile Synthesis

Yanyan Cui, Xinmiao Liang, Jingchao Chai, Zili Cui, Qinglei Wang, Weisheng He, Xiaochen Liu, Zhihong Liu, Guanglei Cui,* and Jiwen Feng*

Copyright WILEY-VCH Verlag GmbH & Co. KGaA, 69469 Weinheim, Germany, 2016.

Supporting Information

High performance solid polymer electrolytes for rechargeable batteries:

A self-catalyzed strategy towards facile synthesis

Yanyan Cui, Xinmiao Liang, Jingchao Chai, Zili Cui, Qinglei Wang, Weisheng He, Xiaochen Liu, Zhihong Liu , Guanglei Cui* , Jiwen Feng*

Y. Cui, Dr. J. Chai, Dr. Z. Cui, W. He, Dr. Q. Wang, X. Liu, Prof. Z. Liu , Prof. G. Cui

Qingdao Industrial Energy Storage Research Institute, Qingdao Institute of Bioenergy and Bioprocess Technology, Chinese Academy of Sciences, Qingdao 266101, P. R. China.

E-mail: liuzh@qibebt.ac.cn; cuigl@qibebt.ac.cn

Dr. X. Liang, Prof. J. Feng

State key Laboratory of Magnetic Resonance and Atomic and Molecular Physics, Wuhan Institute of Physics and Mathematics, Chinese Academy of Sciences, Wuhan 430071, P. R. China.

Dr. X. Liang, Dr. J. Chai, Dr. Z. Cui, Dr. Q. Wang, Prof. Z. Liu , Prof. G. Cui, Prof. J. Feng

University of Chinese Academy of Sciences, No. 19A Yuquan Road, 100049, Beijing, P. R. China.

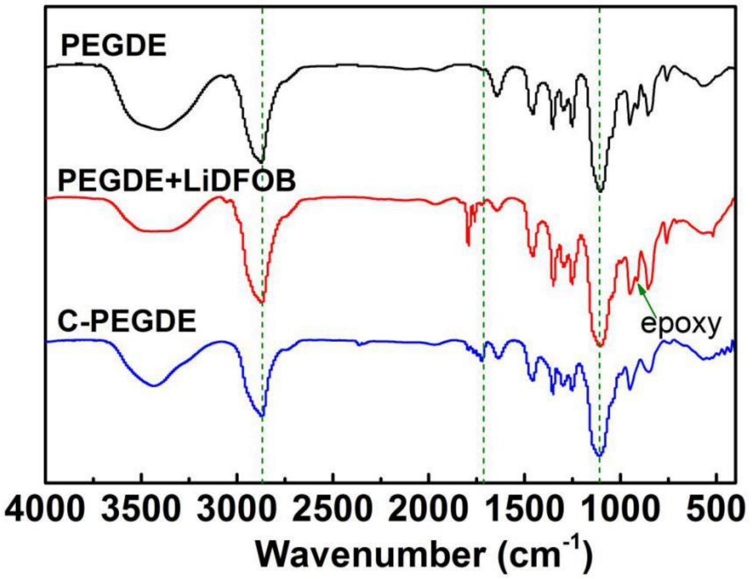

Figure S1. FTIR spectra of the C-PEGDE, precursor solution and PEGDE

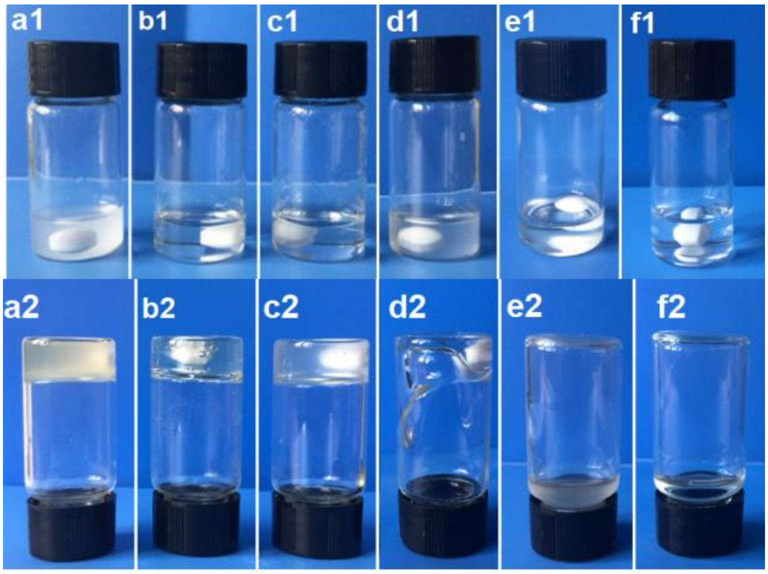

Figure S2. Digital images of solution PEGDE /lithium salt mixture (a1, $LiPF_6$; b1, $LiClO_4$; c1, $LiBF_4$; d1, LiTFSI; e1; LiBOB; f1, none) and solid polymer electrolyte after storage at 80℃ for several hours (a2, b2 c2, d2, e2, f2)

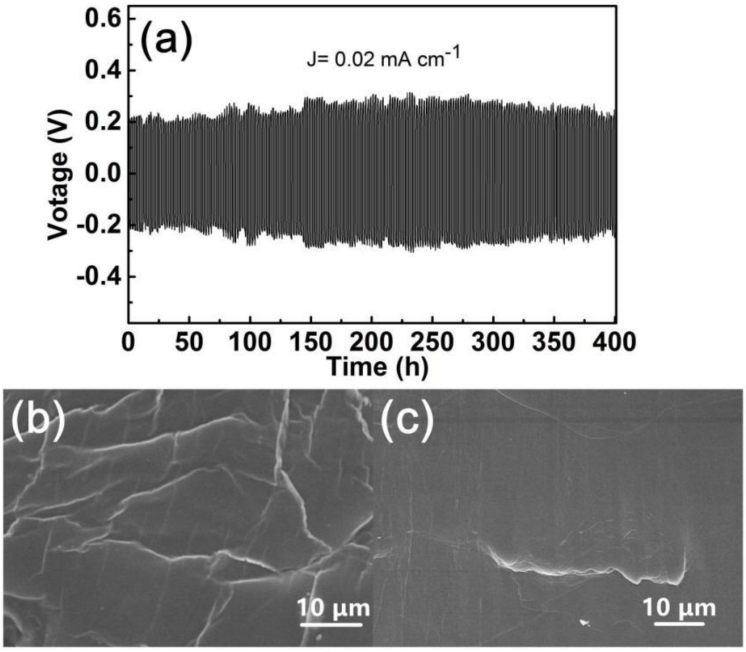

Figure S3. (a) Chronopotentiometry results of Li/C-PEGDE/Li symmetrical cells at room temperature at the current density of 0.02 mA cm^{-2}; (b) Typical SEM images of the surface morphology lithium metal foil after 400 h polarization (c) and the pristine lithium metal foil (d).

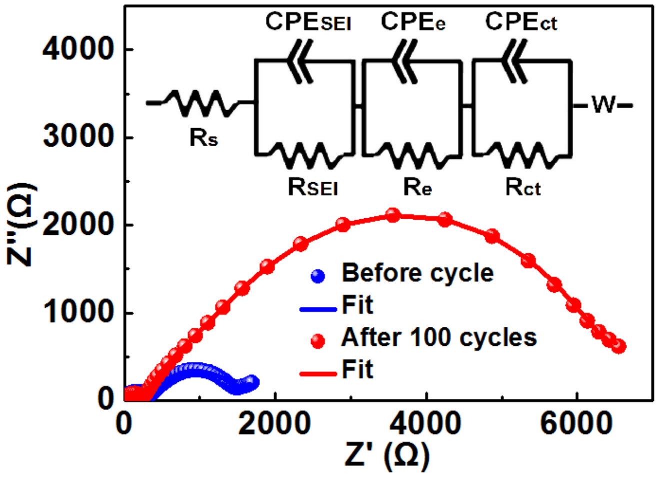

Figure S4. Nyquist impedance plots of composite C-PEGDE based electrolytes. The insert panel shows the equivalent circuit used for fitting the impedance data.

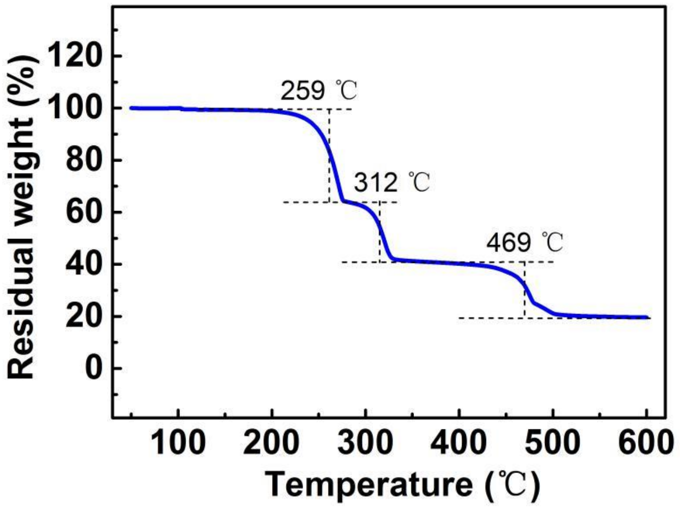

Figure S5. The TGA curve of solid LiDFOB

Table S1. The values of fitting parameters of ionic conductivity of composite C-PEGDE electrolyte membrane.

Sample	A	E_a	T_0
Composite C-PEGDE	7.8×10^{-3} S cm^{-1} K$^{1/2}$	2.09 KJ mol^{-1}	231.21 K
